# Iron oxide and various metal oxide nanotubes engineered by one-pot double galvanic replacement based on reduction potential hierarchy of metal templates and ion precursors†

**DOI:** 10.1039/d0ra07482a

**Published:** 2020-10-20

**Authors:** Aloka Paragodaarachchi, Steven Medvedovsky, Justin Fang, Timothy Lau, Hiroshi Matsui

**Affiliations:** a Department of Chemistry, Hunter College, City University of New York, 695 Park Avenue, New York, NY 10065, USA.; b PhD Program in Chemistry, The Graduate Center of the City University of New York, New York, NY 10016, USA; c PhD Program in Biochemistry, The Graduate Center of the City University of New York, New York, NY 10016, USA; d Department of Biochemistry, Weill Cornell Medicine, 413 East 69^th^ Street, New York, NY 10021, USA

## Abstract

A one-pot double galvanic approach was explored for the rational synthesis of metal oxide nanotubes, predictable based on the reduction potential hierarchy of templates and ion precursors (*e.g.*, Ag nanowire substrate is oxidized by MnO_4_^−^ ions and it is consecutively reduced by Fe^2+^ ions to form an Fe_2_O_3_ nanotube). This method generated a variety of metal oxide nanotubes *via* a redox potential landscape.

A variety of inorganic nanoparticles with different shapes and sizes have been synthesized since these parameters could be used for fine-tuning their physical and biological properties.^1^ Among them, one dimensional (1D) nanostructures are pivotal due to their characteristic electrical, magnetic and chemical properties.^2,3^ Free standing hollow 1D metal oxide nanoparticles represent an intriguing class of nanomaterials due to their high surface to volume ratio and low density,^4,5^ leading to distinct properties in catalysis,^6^ energy storage,^7^ gas sensors,^8^ photodetectors,^2^ and in biomedical applications such as drug delivery.^9^

Previously, metal oxide nanotubes were synthesized by techniques such as template-mediated and template-free methods which include hydrothermal reaction, and chemical etching method.^4,10^ In the template-mediated method, post-treatment is necessary to remove templates. Thus, it adds complexity to the synthesis process, increases frequency of structural deformation, and introduces more impurities even after undergoing a separation process for the removal of leftover template in the sample.^7^ Moreover, the size of the nanotube is also limited by the size of the template, which is often larger than 200 nm.^5^ Hydrothermal synthesis of metal oxide nanotubes has disadvantages such as difficulty of controlling structure in high aspect ratios.^11–13^ The chemical etching method typically requires specialized equipment to react the precursors in high temperature, controlled atmosphere and pressure conditions, increasing production costs.^14^

One of the most effective and versatile sacrificial template methods for synthesizing hollow metal nanoparticles with controllable composition and size is galvanic replacement.^6,15^ Galvanic replacement is a corrosion process that is driven by the difference in reduction potentials between a metallic substrate and metal ions in solution.^1^ The advantage of utilizing the galvanic mechanism for nanomaterial synthesis is that the resulting hollow nanoparticles can be rationally designed by using precursors that have staggered reduction potentials in their exchange reactions. Upon contact between such metal substrate and ions in solution, the one with lower reduction potential is oxidized, while the other with higher reduction potential is reduced, and as a result, metal ions from solution are plated onto the template.^1,14^

As these metals are exchanged, the final product typically possesses a shape similar to the original substrate, but the element of substrate is then swapped to metal ions from solution. Recently, element exchange triggered by surface energy difference has been applied to engineer various hollow metal nanoparticles,^16^ and Xia group has pioneered the application of galvanic exchange reactions in the synthesis of Au, Pt and Pd hollow metal nanoparticles from Ag nanoparticle substrates.^17,18^ However, this approach has rarely been applied for the synthesis of 1D metal oxide hollow nanostructures starting from sacrificial silver nanowire substrates.

Here, we demonstrate for the first time the synthesis of hollow iron oxide nanotubes starting from silver nanowires using one-pot, two-step galvanic replacement reactions. It should be noted that direct replacement of Ag to Fe_2_O_3_ is not viable considering both species need to oxidize simultaneously, and that is our motivation for developing the one-pot, double galvanic exchange reactions of nanotubes. This method can also be generalized for rational synthesis of other metal oxide nanotubes as the formation is predicted by redox potentials of involved ion species.

Fig. 1 summarizes the synthesis scheme of iron oxide nanotubes starting from silver nanowires as substrate. Here, it should be noted that the reactions are broken down into two steps for the ease of understanding the reaction mechanism, while all of these reactions are done in one pot. The galvanic replacement reaction is driven by the differences in reduction potentials between the two species involved.^15^ In Fig. 1a, Ag is dissolved into solution and electrons through Ag oxidation reduce MnO_4_^−^ ions in solution to Mn_3_O_4_. The replacement to Mn_3_O_4_ in nanotubes takes place due to the redox potential landscape between Ag and MnO_4_^−^ (Fig. 1c) since the absolute value of reduction potential of MnO_4_^−^ (1.47 V) is larger than the one for Ag (−0.8 V) as shown in Fig. 1c.^19^ This first galvanic replacement reaction was initiated by mixing silver nanowires (5 mg mL^−1^, 3 mL) of 20 nm in diameter dispersed in water with an aqueous solution of potassium permanganate (1 mM, 18.95 mL) and subsequently heating the reaction mixture to 100 °C for 40 minutes.^19^ At this point, the shape of the original nanowire (Fig. 2a) has already transformed into a hollow nanotube (Fig. 2b).

In the second galvanic exchange reaction (Fig. 1b), Mn_3_O_4_ nanotubes from galvanic reaction 1 are transformed to Fe_2_O_3_ nanotubes as Mn ions are replaced by Fe ions. The redox potential difference is aligned favourably for the oxidation of Fe ions (−0.77 V) *via* the reduction of Mn ions (1.82 V) (Fig. 1c) during this process. Fe_2_O_3_ nanotubes were generated when Mn_3_O_4_ nanotubes were mixed with iron(II) perchlorate solution (1 mg mL^−1^, 12 mL) at 80 °C for 2 hours. The resulting sample is then centrifuged at 3000*g* for 10 minutes, and the pellet is resuspended in deionized water for analysis. Transmission electron microscopic (TEM) image of Fe_2_O_3_ nanotubes (Fig. 2c) reveals that the diameter of nanotubes increases from 27.0 ± 2.0 nm in Mn_3_O_4_ nanotubes to 40.0 ± 3.0 nm in Fe_2_O_3_ nanotubes. The elemental mapping of energy-dispersive X-ray spectroscopy (EDXS) was also applied to analyze the elemental replacement in the process of one-pot galvanic reaction in Fig. 1. When intermediate products of nanotubes after galvanic reaction 1 (Fig. 3a) were probed by EDXS mapping with scanning transmission electron microscopy (STEM), the composition was mostly Mn (Fig. 3c) and O (Fig. 3d) with minimal leftover Ag substrate (Fig. 3b). The second galvanic exchange reaction generated Fe_2_O_3_ nanotubes (Fig. 3e) in high yield and Mn ions were successfully exchanged by Fe ions (Fig. 3i), and the resulting nanotubes were almost free from Ag (Fig. 3f) and Mn (Fig. 3g).

When the surface structure of Mn_3_O_4_ nanotubes was examined by high resolution TEM (HRTEM), pinholes on the sidewalls of Mn_3_O_4_ nanotubes were revealed where the dark contrast of remaining Ag inside make these holes more visible (inside red circles in Fig. 2d). These pinholes are even more evident in a HRTEM image in Fig. 2e. This observation led to the hypothesis that the hollowing process of template nanowires could occur through the pinhole dissolution mechanism. Pinhole corrosion is the process where pinholes serve as paths of material transport during the dissolution of the core of template nanoparticles in the course of the redox reactions.^15^ In this mechanism, the stoichiometry of redox reaction is important to determine the degree of atomic replacement and hollowing process which in turn affects the nanotube’s composition and sidewall thickness.^20^ In the hollowing process of Ag nanowires, the Ag → Mn replacement reaction is;

$$16{{\text{H}}_{\left(\text{aq}\right)}}^{+}+{3{\text{MnO}}_{4(\text{aq})}}^{-}+13{\text{Ag}}_{\left(\text{s}\right)}\to {\text{Mn}}_{3}{\text{O}}_{4(\text{s})}+{13{\text{Ag}}_{\left(\text{aq}\right)}}^{+}+8{\text{H}}_{2}{\text{O}}_{\left(l\right)}$$

In this reaction, one Mn_3_O_4_ is produced at the expense of 13Ag atoms based on the stoichiometric ratio above. Such high turnover of Ag replacement through the efficient pinhole dissolution paths should guide the redox reaction to nearly complete replacement, which is difficult to be accomplished solely by room temperature Kirkendall effect (*i.e.*, the diffusion of Ag from template nanowires is slow at relatively low reaction temperature).^21^ In addition, the atomic% ratio of Ag *versus* Mn of the Mn_3_O_4_ nanotubes is 0.1 : 1.0 by the STEM-EDX mappings of Fig. 3b and c, showing efficient Ag atom replacement. This result also supports that the transformation from Ag nanowire to Mn_3_O_4_ nanotubes is mainly driven by the pinhole dissolution mechanism and that the Kirkendall effect may play only a minor role in the replacement reaction.^15^ Based on TEM images, the wall thickness of Mn_3_O_4_ nanotubes is 5.1 ± 2.0 nm (*n* = 23) (Fig. 2b) after Ag nanowire templates with diameters of 20 ± 2.0 nm (Fig. 2a) are consumed, which is consistent with the previous observations about the degree of sidewall thickness with respect to the diameter of templates with similar stoichiometry in their replacement reactions.^20,22^ Further atomic replacement from Mn to Fe occurs by the reaction;

$${8{\text{H}}_{\left(\text{aq}\right)}}^{+}+{\text{Mn}}_{3}{\text{O}}_{4(\text{s})}+{2{\text{Fe}}_{\left(\text{aq}\right)}}^{2+}\to {3{\text{Mn}}_{\left(\text{aq}\right)}}^{2+}+4{\text{H}}_{2}{\text{O}}_{\left(\text{l}\right)}+{2{\text{Fe}}_{\left(\text{s}\right)}}^{3+}$$

where two Fe ions are produced by replacing one Mn_3_O_4_. Since numerous lattice vacancies would be formed on the sidewall of Mn_3_O_4_ nanotubes (due to high atomic replacement ratio of Ag-to-Mn in 13 : 1) and the possibility of these vacancies to coalesce to form pinholes,^23^ the replacement from Mn to Fe is efficient through pinholes. Such processes will further deplete the amount of Ag in the resulting Fe_2_O_3_ nanotubes, thus, it is reasonable to observe almost complete replacement of Ag in Fe_2_O_3_ nanotubes with atomic% ratio of Ag *versus* Fe of 0.006 : 1 from Fig. 3f and i. Moreover, due to the atomic ratio of this replacement, it is plausible to observe the wall thickness of Fe_2_O_3_ nanotubes to increase to 11.2 ± 3.2 nm (*n* = 23) (Fig. 2c).

Another supporting observation for the pinhole dissolution mechanism is that the volume of voids inside Ag nanowires increases during the transformation to Mn_3_O_4_ nanotubes with the concentration of Mn precursor. When various concentrations of KMnO_4_ were reacted with Ag nanowire solution, a series of TEM images in Fig. S1† showed that void formation is expanded by increasing the amount of KMnO_4_. Since more Mn ions consume Ag ions with efficient atomic replacement through pinholes, these TEM images support the pinhole dissolution mechanism as a major pathway. In summary, all evidence here supports that pinhole dissolution is the main mechanism for the synthesis of Fe_2_O_3_ nanotubes from Ag nanowire templates.

Since manganese possesses a very high reduction potential of 1.82 V, it is feasible to be utilized as a template for a variety of metal oxide nanotubes. Thus, this enables the design of a variety of metal oxide nanotube syntheses, as long as the replacing metal ions can be oxidized and have reduction potentials smaller than 1.82 V (in absolute values). Due to the high reduction potential of Mn ions, there are many metal ions that align with this reduction potential hierarchy for the second galvanic replacement as shown in Fig. 1c. To test the generality of this one-pot, double galvanic method for metal oxide nanotube synthesis, we examined the synthesis of NiO_2_, CuO, and SnO_2_ nanotubes from Ag nanowire substrates. In terms of the redox potential landscape, all of the reduction potentials are appropriate for one-pot double galvanic exchange as the reduction potential of NiO_2_/Ni^2+^ , SnO_2_ /Sn^2+^ and Cu^2+^/Cu^+^ is 1.68 V, −0.094 V, and 0.161 V, respectively.^24,25^ In theory, these ions can reduce Mn_3_O_4_ (1.82 V) during the second galvanic replacement reaction because their reduction potentials in absolute values are lower than Mn_3_O_4_. In this trial, tin(II) chloride, copper(I) chloride, and nickel(II) chloride were used as precursor solutions to synthesize SnO_2_, CuO, and NiO_2_ nanotubes. TEM images (Fig. 4a, d and g) indicate that all of these nanotubes possess a hollow structure with an average diameter of 40 nm, suggesting that their size was controlled by the size of Ag nanowire substrate. The elemental transformation of template nanowires to target nanotubes was confirmed by EDXS maps in Fig. 4b, c, e, f, h and i.

In conclusion, we combined two galvanic reactions in one-pot for the synthesis of iron oxide nanotubes from commercially available Ag nanowires. The large reduction potential of the Mn_3_O_4_ nanotube intermediate opens this method to final exchange by various metal ions to form many different metal oxide nanotubes. We have demonstrated it by the synthesis of other metal oxide nanotubes such as SnO_2_, CuO, and NiO_2_, which have favorable staggered redox potential arrangement with Mn_3_O_4_ reduction. This generalized double galvanic replacement approach will offer robust, economical, and scale-up engineering of a variety of hollow one-dimensional metal oxide nanostructures.

## Supplementary Material

Supplement

## Figures and Tables

**Fig. 1:**
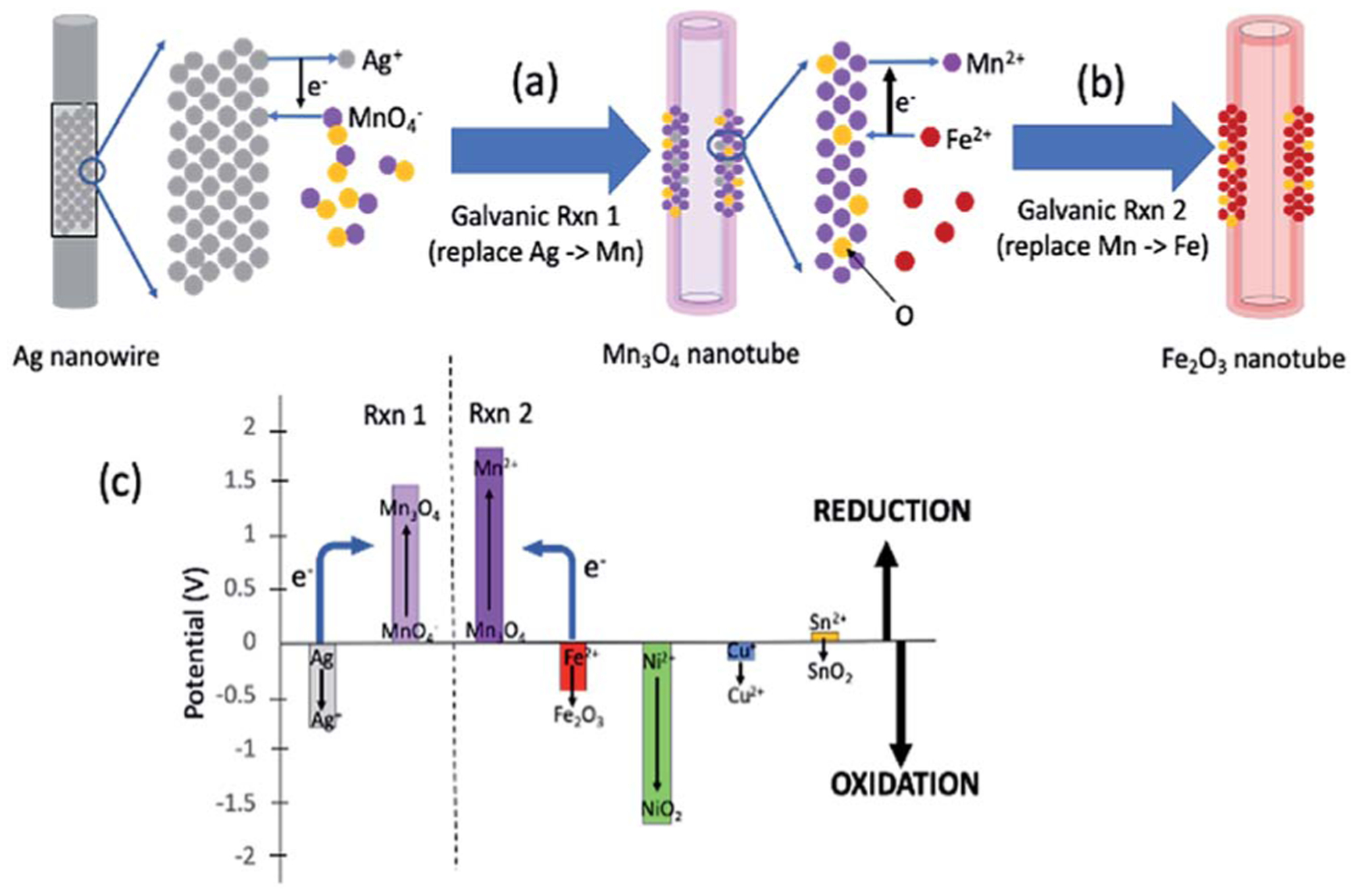
General concept of one-pot double galvanic exchange reactions for the synthesis of iron oxide nanotubes. (a) The first galvanic exchange reaction to generate Mn_3_O_4_ nanotubes from Ag nanowire substrate. (b) The second galvanic exchange reaction to replace Mn to Fe nanotubes. (c) Reduction potential landscape for iron oxide and other possible metal oxide in nanotube formation (half-reactions and their electric reduction potentials are also shown in ESI Table S-1†). When the reduction potential of one element is larger than the other, the element with lower reduction potential (*e.g.*, Fe) can donate its electrons to reduce Mn_3_O_4_, which has a larger reduction potential (see blue arrows for the direction of electron flow). A variety of metal oxide nanotubes in addition to iron oxide can be formed when the final element of the nanotube is oxidized due to the large reduction potential of the intermediate Mn_3_O_4_ nanotube.

**Fig. 2:**
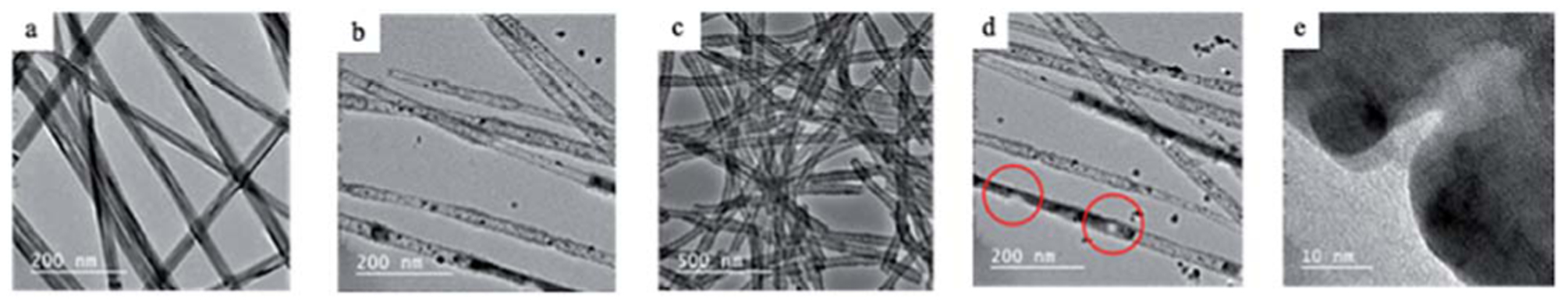
TEM images of (a) Ag nanowire substrates, (b) Mn_3_O_4_ nanotubes, (c) Fe_2_O_3_ nanotubes, (d) a section of Mn_3_O_4_ nanotubes where pinholes were observed on the surface (red circles), (e) pinholes in (d) with a higher magnification.

**Fig. 3:**
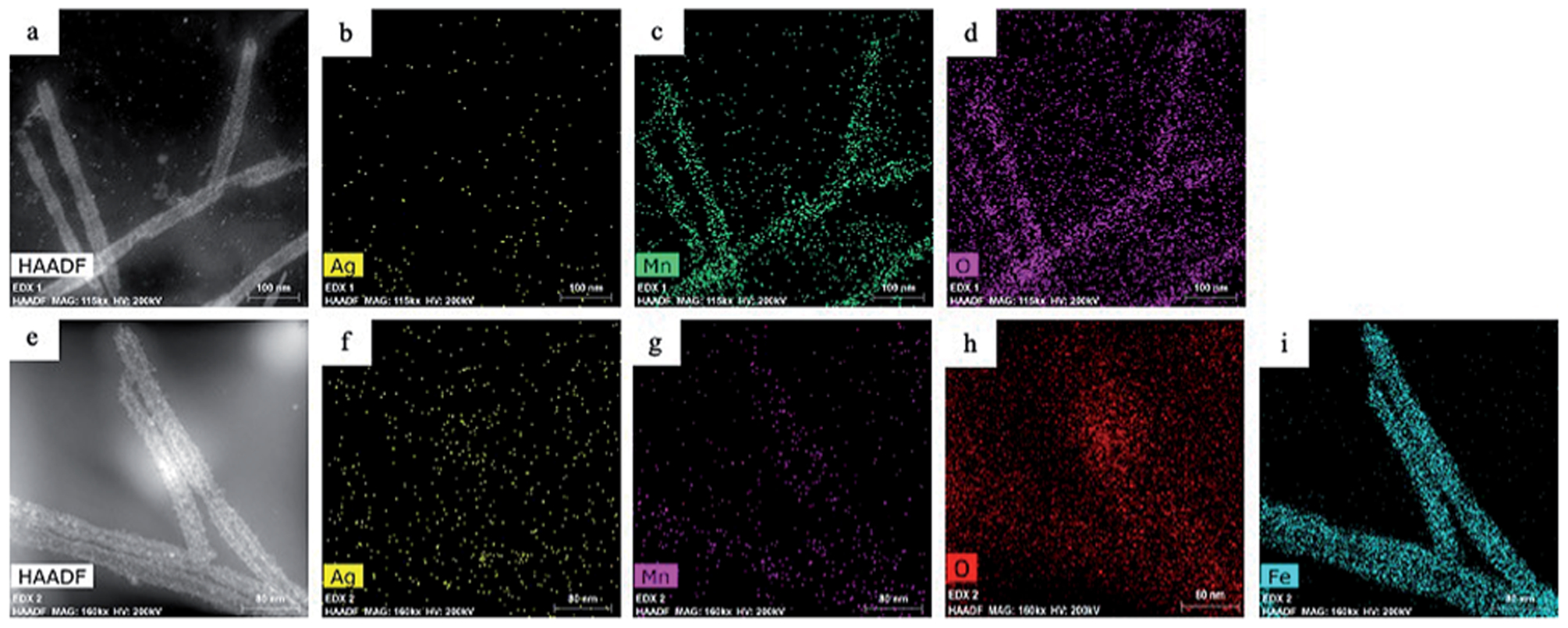
(a) Scanning transmission electron microscopic (STEM) image of Mn_3_O_4_ nanotubes and corresponding elemental mapping in (b) Ag,(c) Mn, and (d) O with EDXS. (e) STEM image of Fe_2_O_3_ nanotubes and corresponding elemental mapping in (f) Ag, (g) Mn, (h) O, and (i) Fe with EDXS.

**Fig. 4:**
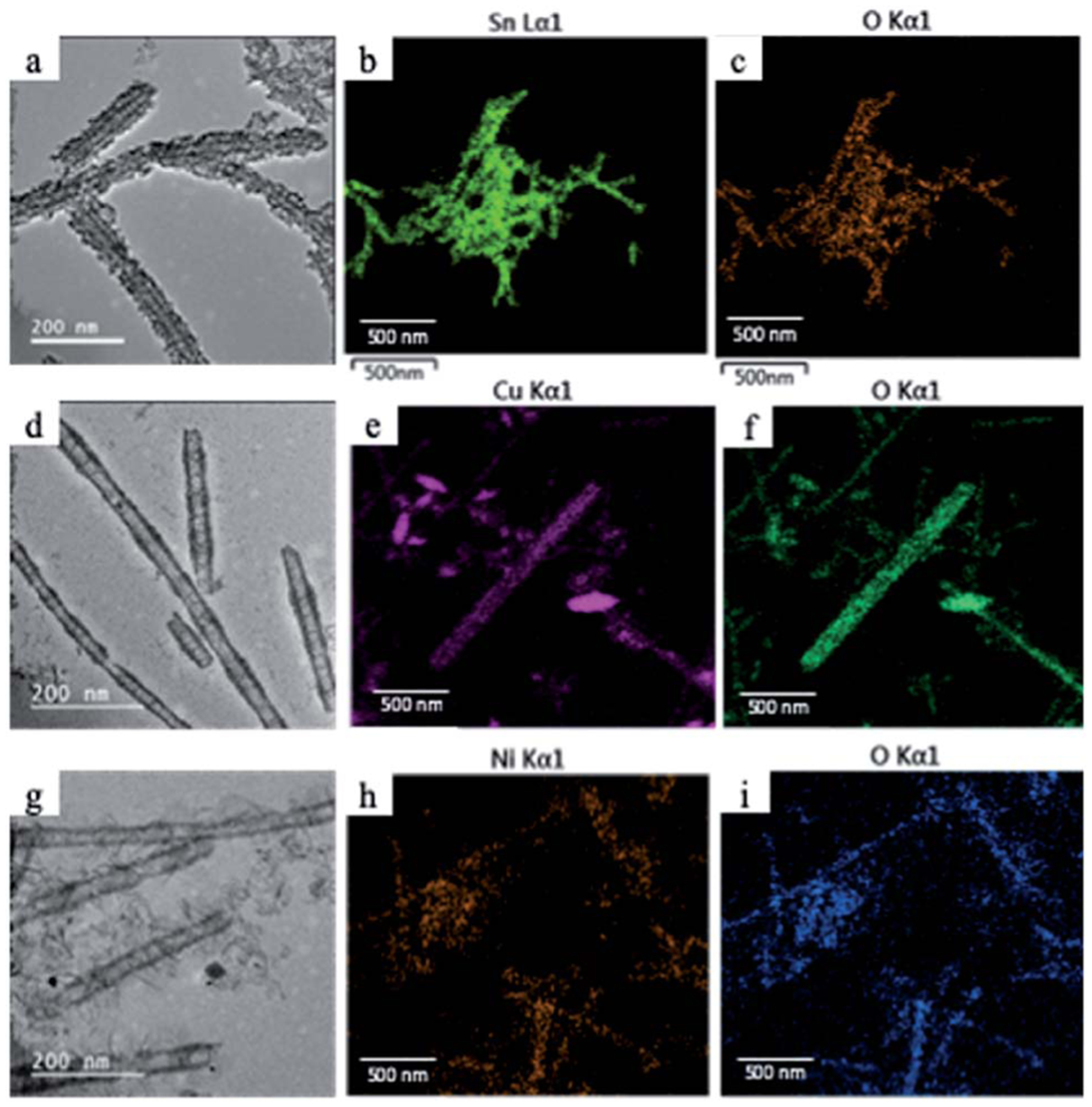
(a) TEM image of SnO_2_ nanotubes and elemental mapping of (b) Sn and (c) O with EDXS. (d) TEM image of CuO nanotubes and elemental mapping of (e) Cu and (f) O with EDXS. (g) TEM image of NiO_2_ nanotubes and elemental mapping of (h) Ni and (i) O with EDXS.

## Footnotes

† Electronic supplementary information (ESI) available. See DOI:10.1039/d0ra07482a
