# Supplementary material for: Iron oxide and various metal oxide nanotubes engineered by one-pot double galvanic replacement based on reduction potential hierarchy of metal templates and ion precursors
Source: RSC Adv. Author manuscript; Available in PMC 2021 Apr 20. (PMC8057675; doi:10.1039/d0ra07482a)
Supplement: Supplementary file 1 [file NIHMS1685824-supplement-Supplement.pdf]

Electronic Supplementary Information

Iron Oxide and Various Metal Oxide Nanotubes Engineered by One-Pot Double Galvanic Replacement Based on Reductiob Potential Hierarchy of Metal Templates and Ion Precursors

Aloka Paragodaarachchi,[a,b], Steven Medvedovsky,[a] Justin Fang,[a,b] Timothy Lau,[a] and Hiroshi Matsui [a, b, c, d *]

a. Department of Chemistry, Hunter College, City University of New York, 695 Park Avenue, New York, NY 10065 (USA)

b. Ph.D. Program in Chemistry, The Graduate Center of the City University of New York, New York, NY 10016 (USA)

c. Ph.D. Program in Biochemistry, The Graduate Center of the City University of New York, New York, NY 10016 (USA)

d. Department of Biochemistry, Weill Cornell Medical College, 413 East 69th Street, New York, NY 10021 (USA)

Experiments

Materials:
Silver nanowires, diam. × L 20 nm (± 2 nm) × 12 µm (± 2 µm) 5 mg/mL (in water), Polyvinylpyrrolidone (PVP, Mw ~ 55,000) and Tin(II) chloride were purchased from Sigma-Aldrich. Iron(II) perchlorate hydrate was purchased from Alfa Aesar. Potassium permanganate, Copper(I) chloride and Nickel(II) chloride were purchased from Fisher Scientific.

Mn_3O_4 Nanotube Synthesis: Manganese oxide nanotubes were synthesized by a modified version of a previously published method.[1] 3 mL of silver nanowires, 2.75 mL of 0.3 mM PVP (aq) and 18.95 mL of 1 mM $KMnO_4$ were added to a three-necked 50 mL flask with a reflux condenser. The flask was heated to 100 ^oC in air under magnetic stirring and left to react for 40 minutes, producing Mn_3O_4 nanotubes. To perform a second galvanic replacement, no additional steps are needed. To reserve Mn_3O_4 nanotubes, the solution was removed from heating and left to cool to room temperature. After cooling, the solution was centrifuged at 3000 G for 10 minutes and the supernatant was discarded. The pellet was then resuspended in deionized water.

Fe_2O_3 Nanotube Synthesis: The temperature of the Mn_3O_4 nanotube reaction mixture was lowered to 80 ^oC. Then, 12 mL of 1 mg/mL aqueous iron(II) perchlorate solution was added to the flask. Heating continued at 80 ^oC in air under magnetic stirring for 120 minutes, producing Fe_2O_3 nanotubes. After cooling, the solution was centrifuged at 3000 G for 10 minutes and the supernatant was discarded. The pellet was then resuspended in deionized water.

SnO2 Nanotube Synthesis: First, 4 mg of tin(II) chloride was dissolved in 15.8 ml of 5M HCl and 2.0 µl of deionized water. Then, 3 mL of the Mn_3O_4 nanotube reaction mixture was transferred to a different 50 mL three-neck flask equipped with a reflux condenser. The flask was heated to 90 ^oC in air under magnetic stirring, and 5.0 ml of the tin(II) chloride solution was added. Heating continued at 90 ^oC for 90 minutes, producing SnO_2 nanotubes.

CuO Nanotube Synthesis: First, 3 mg of copper(I) chloride was dissolved in 50 mL of deionized water. Then, 3 mL of the Mn_3O_4 nanotube reaction mixture was transferred to a different 50 mL three-neck flask equipped with a reflux condenser. The flask was heated to 90 ^oC in air under magnetic stirring, and 20.0 ml of the copper(I) chloride solution was added. Heating continued at 90 ^oC for 90 minutes, producing CuO nanotubes.

NiO_2 Nanotube Synthesis: First, 20 mg of nickel(II) chloride was dissolved in 1 mL of deionized water. Then, 3 mL of the Mn_3O_4 nanotube reaction mixture was transferred to a different 50 mL three-neck flask equipped with a reflux condenser. The flask was heated to 90

^{o}C in air under magnetic stirring, and 20.0 ml of the nickel(II) chloride solution was added. Heating continued at 90 ^{o}C for 90 minutes, producing NiO_2 nanotubes.

Microscopy and Elemental Analysis
Transmission electron micrographs were taken using a JEOL JEM-2100 TEM at 200 kV with a LaB6 gun. Images were captured using 4GB Gatan ultrascan camera model 994 US 1000XP and Digital Micrograph V. 2.1. For the elemental mapping, scanning transmission electron microscope (STEM,) and energy-dispersive X-ray spectroscopy (EDXS) were carried out in a FEI-Titan Themis 200 TEM operating at 200 kV equipped with a 4k x 4k Ceta 16M CMOS camera. A high angle angular dark field (HAADF) detector was used for STEM and Windowless Super-X EDS detector system with ESPRIT software was applied for EDXS.

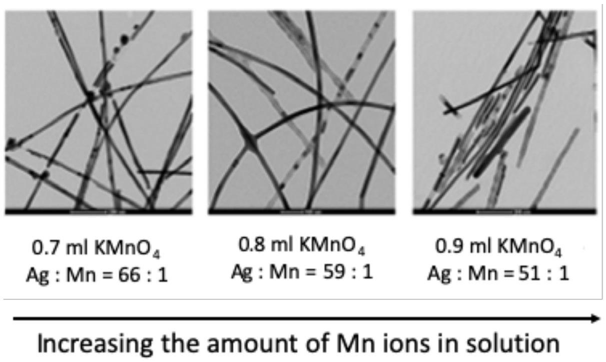

Figure S-1. TEM images of Mn_3O_4 nanotubes synthesized in Ag nanowire solution (5 mg/ml) containing various amounts of Mn ions. 0.7 ml, 0.8 ml, and 0.9 ml (from left to right) of $KMnO_4$ (1 mM) was added to 1 ml of Ag nanowire solution.

Half Reaction	Electric Reduction Potential (V)
$Ag^{+}_{(aq)} \rightarrow Ag_{(s)}$	0.8 [1]
$MnO_{4}^{-}{}_{(aq)} \rightarrow Mn_3O_{4(s)}$	1.47 [2]
$Mn_3O_{4(s)} \rightarrow Mn^{2+}_{(aq)}$	1.82 [3]
$Fe^{3+}_{(s)} \rightarrow Fe^{2+}_{(aq)}$	0.77 [3]
$NiO_{2(s)} \rightarrow Ni^{2+}_{(aq)}$	1.68 [4]
$Cu^{2+}_{(s)} \rightarrow Cu^{+}_{(aq)}$	0.0161 [5]
$Sn^{4+}_{(s)} \rightarrow Sn^{2+}_{(aq)}$	-0.094 [5]

Table S1. A breakdown of the half-reactions in the reduction potential landscape in Figure 1-(c).

References

1. Y. G. Sun, B. T. Mayers and Y. N. Xia, *Nano Lett.*, 2002, **2**, 481-485.
2. H. Dong, E. K. Koh and S. Y. Lee, *J. Nanosci. Nanotechnol.* , 2009, **9**, 6511–6517
3. M. H. Oh, T. Yu, S.-H. Yu, B. Lim, K.-T. Ko, M.-G. Willinger, D.-H. Seo, B. H. Kim, M. G. Cho, j.-H. Park, K. Kang, Y.-E. Sung, N. Pinna and T. Hyeon, *Science*, 2013, **340**, 964-968.
4. S. G. Bratsch, *J. Phys. Chem. Ref. Data* 1989, **18**, 1-21.
5. Bard, R. Parsons and J. Jordan, *Standard potentials in aqueous solution*, Marcel Dekker, Inc., New York, 1985.
